# Splenogondal Fusion With Accessory Spleens Identified With Laparoscopy

**DOI:** 10.7759/cureus.9017

**Published:** 2020-07-06

**Authors:** Emily Biben, Jeff Pugach

**Affiliations:** 1 Pediatric Urology, Cook Children's Medical Center, Fort Worth, USA; 2 Pediatric Urology, Cook Children’s Medical Center, Fort Worth, USA

**Keywords:** splenogonadal fusion, testicular mass, accessory spleen, orchiopexy, congenital malformation, cryptorchidism

## Abstract

Splenogonadal fusion (SGF) is a congenital abnormality characterized by fusion between the spleen and gonad. This rare condition is difficult to diagnose preoperatively, and there are fewer than 200 cases documented previously in the literature. This report describes a young male who presented with bilateral cryptorchidism and was diagnosed with SGF and accessory spleens using laparoscopy. SGF is a rare but possible finding to recognize in children with abnormal paratesticular masses, and laparoscopic techniques as demonstrated in this case will increase the ease of diagnosis and early treatment.

## Introduction

Splenogonadal fusion (SGF) is a congenital malformation with fewer than 200 cases documented in the literature since first described by Bostroem in 1883 [1,2]. SGF can be associated with additional abnormalities, most notably limb defects and micrognathia [3]. Due to its rarity, most cases present as cryptorchidism or a palpable mass and are discovered incidentally during orchidopexy. In a previous review of 137 cases, 16.8% were discovered postmortem and 37% of SGF patients underwent orchiectomy due to suspicion of tumor [4]. With increasing use of laparoscopy, we expect that SGF will become readily diagnosed. We present a patient with discontinuous SGF and numerous accessory spleens to raise awareness of the rare but possible findings when encountering abnormal paratesticular masses in children. 

## Case presentation

An otherwise healthy, four-month old male was noted on evaluation to have a right nontender inguinal testis and left nonpalpable testis. Additionally, diagnoses of chordee and hypospadias were made. The patient’s subsequent chromosome analysis showed a normal 46,XY karyotype. At this time, the patient exhibited no additional physical or developmental abnormalities, and further genetic testing was deemed unnecessary.

**Figure 1:**
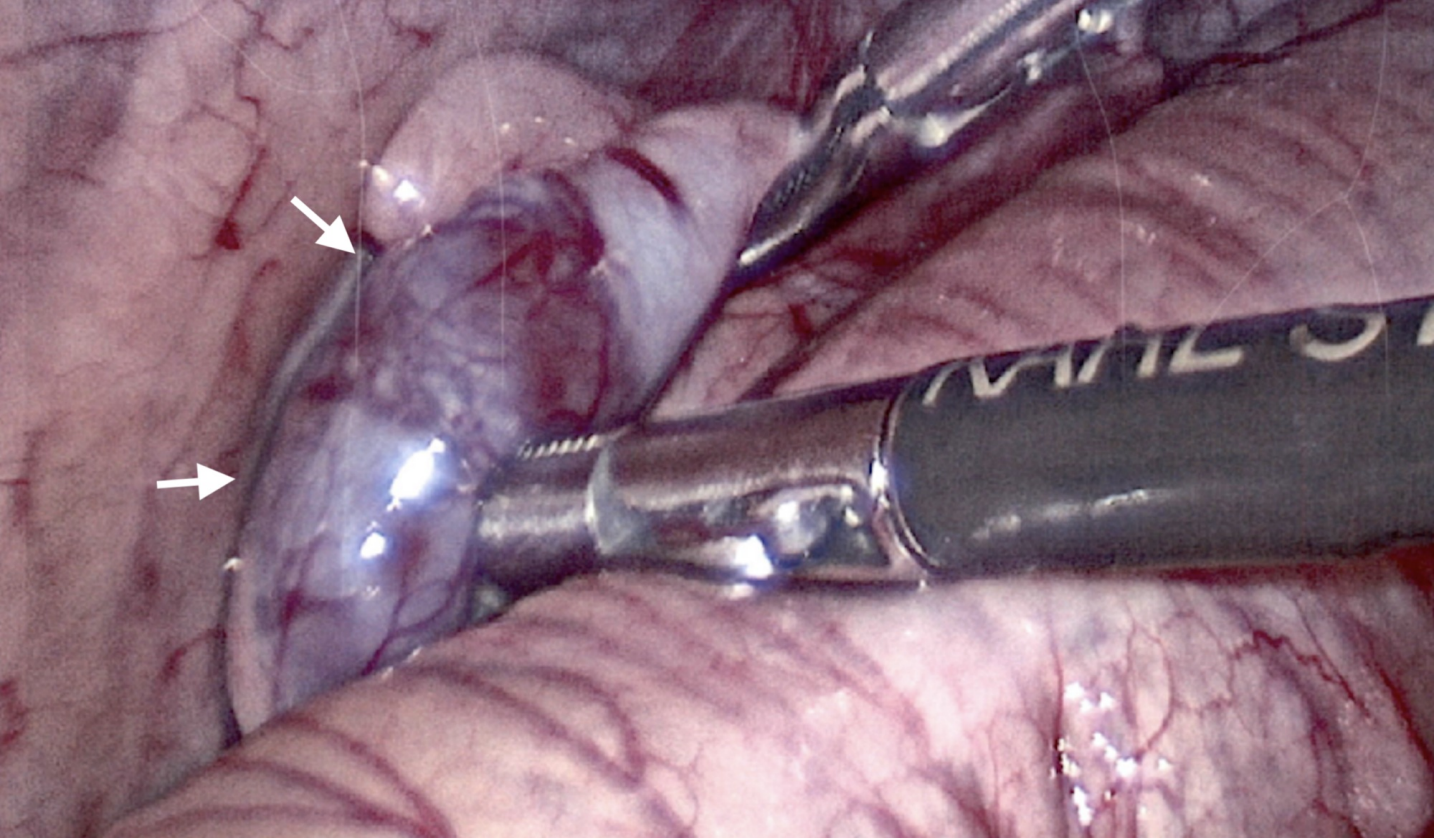
Left abdominal testis in-situ with adherent accessory spleen.

**Figure 2:**
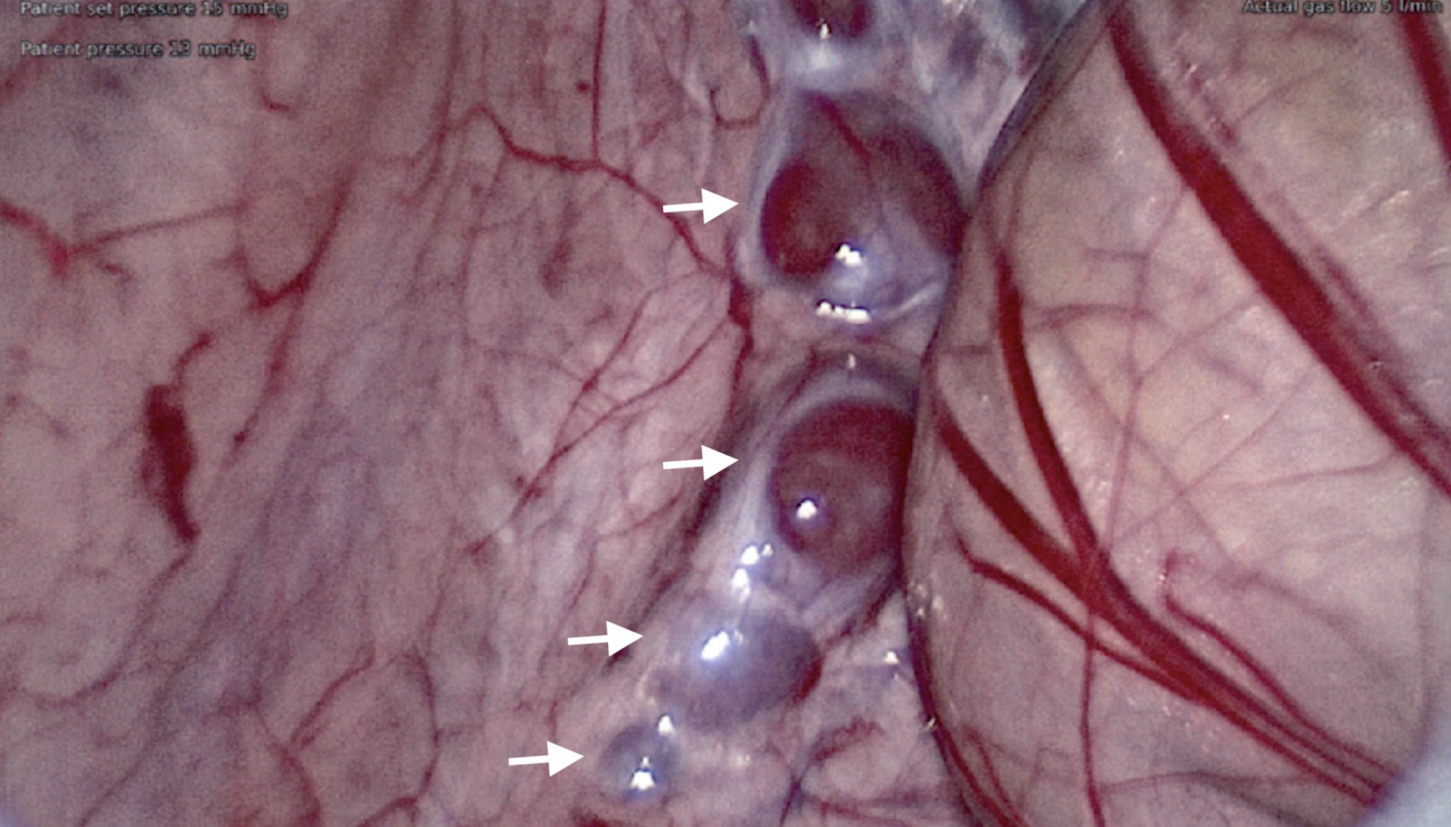
Accessory spleens in left paracolic region.

**Figure 3:**
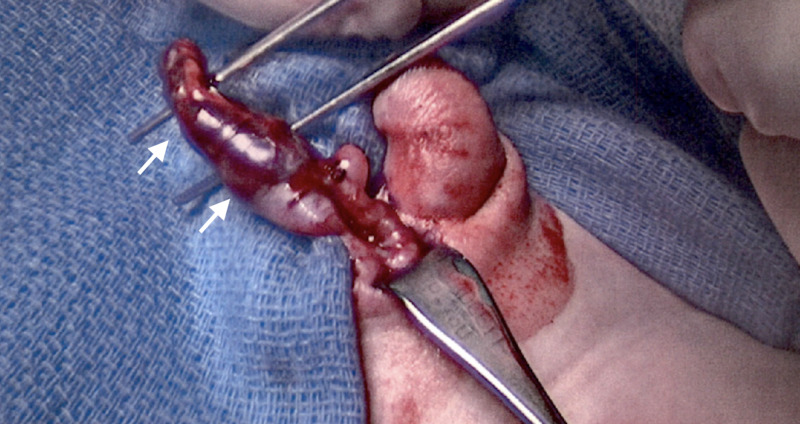
Left testis in scrotal position with adherent accessory spleen.

Diagnostic laparoscopy at five months of age revealed the left testis with an associated accessory spleen away from the internal ring (Figure 1), splenules tracking up the left colic gutter (Figure 2), and a separated epididymis. The patient’s normal spleen was identified with no signs of organ injury. The left testis with associated spleen was mobilized into the scrotum with the vas deferens and gonadal vessels (Figure 3). Open attempts at separation of the structures proved unsuccessful, necessitating orchiectomy, as the association lacked a distinct plane, and the epididymis appeared to enter the splenic tissue. Frozen section confirmed gonadal fusion of an accessory spleen with normal splenic tissue, a rudimentary epididymis, and germ cells within the seminiferous tubules. Standard inguinal right orchidopexy and hypospadias repair were performed without complication. The child followed up four months postoperatively with a normal right testis and well-healed hypospadias.

## Discussion

The spleen forms during weeks 5 to 8 of gestation from mesenchymal progenitors in the dorsal mesogastrium. Simultaneously, the gonadal ridge is developing and can inappropriately fuse to the splenic primordia when gut rotation occurs, resulting in left-sided SGF. SGF is classified into the following types: continuous (55% of cases) when the spleen directly attaches to the gonad via band-like connection and discontinuous (45% of cases) when ectopic splenic tissue connects to the gonad, but no attachment to the normal spleen is identified [3]. Additional congenital abnormalities are most often found in association with the continuous type. It is suggested that an insult occurs during early development, potentially causing accompanying malformations in the limb buds and mandible. Timing of insult may correlate to the severity of defects, yet the mechanism of insult remains unclear. Studies propose a simple adhesion or lack of apoptosis could occur, but teratogenic or inflammatory processes may also be attributed [3,5]. Furthermore, one previous report identified an accessory spleen in a sibling of an SGF patient, raising a genetic component [3].

Current standard treatment of SGF is to remove the uniformly benign splenic mass from the testicle. In the patient described, the testicle was brought down, but unfortunately the lack of a visible plane between the testis and hypervascular accessory spleen necessitated orchiectomy. With no indication of future clinical risk caused by splenules left in the path of testicular descent (Figure 2), intervention of the masses tracking the colic gutter was not required in our patient. Some suggest, when asymptomatic, SGF does not require surgery, but adverse outcomes have been reported in unique cases. Primary infertility was identified in a 25-year-old male with right post-pubertal cryptorchidism and untreated left SGF. Compression of the developing testis by ectopic splenic tissue resulted in a loss of testicular function in adulthood [3]. Furthermore, there are few cases of testicular carcinoma in situ and germ cell tumors reported in adult SGF patients [6]. Cryptorchidism occurring in older stages of life increases the risk of testicular cancer and may explain the malignancies found in SGF patients. 

Males are predominately affected in the identified cases, yet females are likely underdiagnosed due to the challenge of internal gonad examination. Only two cases of SGF in adult females have been identified and reported in the literature [7]. Moreover, SGF should be distinguished from ovarian splenosis, a rarity that occurs following abdominal trauma or splenectomy and can be discovered during salpingo-oophorectomy. SGF is rarely diagnosed preoperatively on diagnostic imaging. Doppler ultrasound of paratesticular masses has led to few diagnoses, but the image is often nonspecific and can mimic rhabdomyosarcoma or embryonal sarcoma, leading to unnecessary invasive procedures [8]. Superior to inguinal exploration, laparoscopic evaluation affords the surgeon greater visualization of the anatomic findings, and we suggest it as a standard in pediatric patients when there is a high degree of suspicion for the diagnosis. Intraoperatively, with laparoscopy, it is easy to identify a testis with a chain of splenules extending to an otherwise normal spleen. These findings readily indicate SGF and malignancy becomes unlikely. Conversely, there is limited exposure with a groin incision. With the growing use of laparoscopy, the potential for a diagnosis of SGF may increase. Abdominal exploration allowed for early diagnosis and treatment in our case. Combined with frozen section, laparoscopy reveals the most complete diagnostic information. In patients where the testis can be separated and preserved, this would yield optimal outcomes through the most noninvasive technique.

## Conclusions

SGF remains of unknown etiology and results in the abnormal association of the spleen and gonad. We report this case of SGF to recommend laparoscopic evaluation for its usefulness during initial diagnosis and attempt in testicular sparing. The rarity of SGF and its ambiguous appearance on imaging often results in the inability to preoperatively diagnose the condition. Surgeons should be prepared for intraoperative discovery and must take the necessary steps to rule out malignancy. Laparoscopy provides the most reliable method to confirm SGF, and in the ideal approach, the accessory spleen should be separated from the testis to preserve normal testicular function. 
